# A Crab Claw-Inspired Stick-Slip Piezoelectric Actuator with Low-Frequency, High-Speed Operation and Autonomous Backward Motion Suppression

**DOI:** 10.3390/s26206365

**Published:** 2026-10-09

**Authors:** Limin Xie, Longji Wang, Xiaoxu Wei, Bing Fang, Yi Fang

**Affiliations:** College of Mechanical and Electrical Engineering, Fujian Agriculture and Forestry University, Fuzhou 350002, China; lucy_min@163.com (L.X.); 52412040038@fafu.edu.cn (L.W.); 52412040024@fafu.edu.cn (X.W.); fangbing008@163.com (B.F.)

**Keywords:** stick-slip actuator, low frequency high speed, autonomous suppression, bi-directional actuation, bionics

## Abstract

At present, piezoelectric actuators featuring both multi-directional output capability and autonomous backward motion suppression are scarce, which limits the application of piezoelectric actuation technology. To address this issue, inspired by the rapid opening-closing switching of crab claws during movement, a novel bionic crab-claw stick-slip piezoelectric actuator is designed based on the stick-slip actuation principle. The actuator adopts a symmetric structure and exhibits bi-directional motion characteristics and is equipped with a flexible hinge amplification mechanism for autonomous backward motion suppression. In this paper, the structural reliability is first verified through mechanical theoretical analysis and the finite element method. Then, an experimental platform is built, and a prototype is fabricated for experimental testing. The results show that the proposed actuator not only has high symmetry in bi-directional output performance but also demonstrates the effectiveness of the backward motion suppression mechanism in the designed comparative experiments. The mechanism can achieve high-speed motion at low frequencies, with a maximum speed of 4.58 mm/s, which exhibits a high speed under comparable dimensions and a lower driving frequency, and the maximum load capacity reaches 0.9 N.

## 1. Introduction

Currently, piezoelectric actuation technology, owing to its characteristics such as micro-scale and nano-scale displacement output, fast response speed, high resolution, and anti-electromagnetic interference [1,2,3], has become a research focus in fields including biomedical equipment, high-precision sensors, micro-precision positioning platforms, and aerospace engineering [4,5,6,7]. The principle of piezoelectric actuation relies on the inverse piezoelectric effect (electrical energy → mechanical energy) of piezoelectric ceramics: the microscopic expansion and contraction motion of piezoelectric ceramics is converted into the macroscopic displacement change in the mover via a piezoelectric actuator [8]. In recent years, to enhance the output performance of piezoelectric actuation for precision instruments, bionic technology is typically integrated with stepping piezoelectric actuators. By simulating biological structures and motion modes, such integration endows the actuators with larger output displacement, compact structure, and stable motion [9,10].

Based on differences in their operating principles, stepping piezoelectric actuators can be classified into three major categories: ultrasonic type [11,12,13], inchworm type [14,15], and stick-slip type [16]. In comparison, bionic stick-slip piezoelectric actuators have attracted researchers’ attention due to their higher output speed, resolution, and load capacity [17,18]. For example, Wang L et al. proposed a stick-slip piezoelectric actuator bionic to human body bending motion, which achieved a maximum output speed of 19.33 mm/s and could output a force of 1.8 N under a preload of 2 N [19]. Yan L et al. designed a stick-slip piezoelectric actuator bionic to the forefoot of a mantis; this actuator outputs rotational displacement, realizing a maximum speed of 4491.38 mrad/s and a minimum rotational resolution of 0.332 μrad [20]. By imitating the structural composition of a flea’s hind legs, Xu M et al. developed a novel bionic parallel piezoelectric stick-slip positioning stage, which can achieve a maximum output speed of 9.03 mm/s, with resolutions of 5 nm (x-direction) and 5.5 nm (y-direction), as well as maximum horizontal and vertical loads of 1.4 N and 40 N, respectively [21].

**Figure 1:**
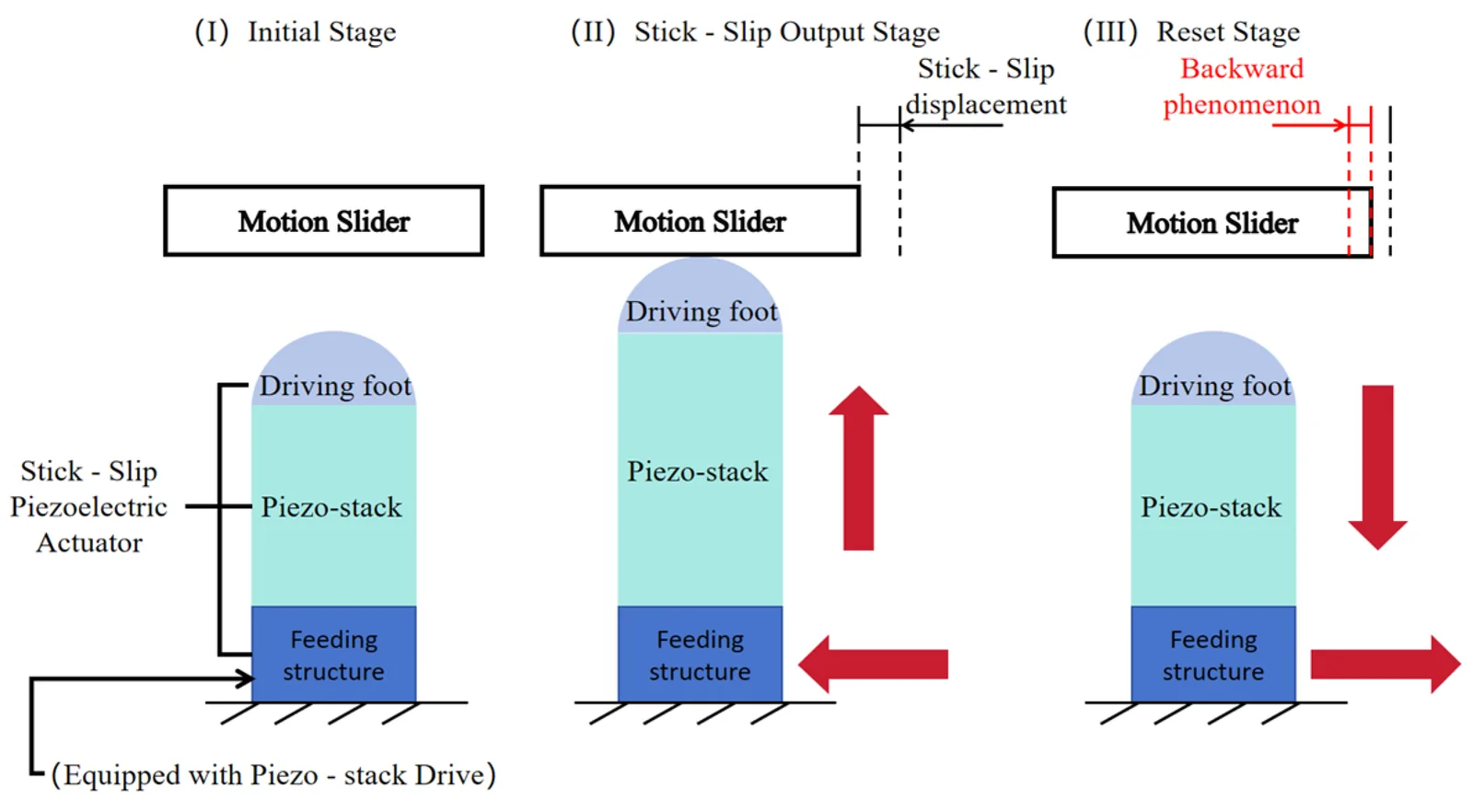
Schematic Diagram of the Motion Principle of the Stick-Slip Piezoelectric Actuator.

Beyond improving output performance, bionics can also be applied to solve the most prominent issue currently existing in stick-slip piezoelectric actuators—the backward-motion phenomenon. The backward motion originates from the friction state transition during the reset stage of the stick-slip cycle: in the retraction phase, the driving foot contracts with the piezoelectric stack, and the contact interface switches from static friction to kinetic friction. Due to inertia mismatch and rapid voltage drop, the driving foot drags the slider to produce a tiny reverse displacement before complete slipping, which reduces effective step displacement and positioning accuracy. A typical stick-slip piezoelectric actuator mainly consists of three components: a driving foot, a stick-slip unit, and a feeding unit, with its driving principle illustrated in Figure 1. At present, this backward-motion phenomenon cannot be completely eliminated, but it can be effectively suppressed through research. Existing suppression strategies can be generally categorized into three types: (1) active preload locking structures, which maintain static friction during reset through normal force control; (2) anisotropic friction surfaces, which generate asymmetric friction coefficients in forward and reverse directions; (3) waveform timing optimization, which adjusts the rising/falling edge rate to match the inertia-friction characteristics. Among them, bionic technology is widely used in the first two routes. There are two primary methods for suppressing backward motion using bionic technology: one is to utilize the coordination between bionic structures and electrical signals. For instance, Liang T et al. designed a butterfly-shaped stick-slip piezoelectric actuator by mimicking the appearance of a butterfly; two identical butterfly-shaped piezoelectric stators were symmetrically mounted on both sides of the mover, and under the alternating stepping mode, sawtooth waveforms with opposite phases, identical periods, and a phase difference were applied as electrical signals. These waveforms enable the driving feet of the bionic structure to generate alternating stepping motion in the positive direction, thereby providing a higher output speed to the rotor while suppressing backward motion [22]; another method is to leverage the properties of bionic biofilms by fabricating them. For example, Liang Y et al. used polymer PDMS (polydimethylsiloxane) to fabricate a film with a surface biological structure similar to that of lemongrass, and placed it between the driving foot of the piezoelectric actuator and the mover. The backward motion was suppressed by the anisotropic frictional characteristics of the surface microstructures [23].

In addition to backward motion, piezoelectric creep is another inherent performance-limiting factor of stick-slip actuators. Piezoelectric creep arises from the slow rotation of polarization domains inside PZT (Lead Zirconate Titanate) ceramics under constant driving voltage, leading to continuous drift of output displacement over time, which is more prominent at low frequency and quasi-static conditions. In this work, we address this issue from two aspects: (1) A trapezoidal driving waveform is adopted, and the flat voltage platform stabilizes domain switching and reduces creep fluctuation; (2) The symmetric flexible hinge structure provides stable preload force, which compensates for preload variation caused by creep.

Meanwhile, compared with the limitation that previous piezoelectric actuators can only output displacement in a single direction, research has gradually shifted toward bi-directional-output stick-slip piezoelectric actuators with broader application scenarios. These actuators can achieve highly symmetric motion in forward and reverse directions, enabling fine positioning adjustments in different directions for instruments in precision positioning platforms, medical fields, etc., thereby improving instrument flexibility and reducing positioning errors [24]. For example, the piezoelectric actuator designed by Wang X et al., which mimics the hindlimb movement of aquatic beetles, can output displacement in two opposite directions by applying positive and negative electrical signals, with maximum no-load speeds of 17.3 mm/s and 20.5 mm/s in the forward and reverse directions, respectively [25]. Yang Z et al. designed a stick-slip piezoelectric actuator by imitating the symmetric structure and movement pattern of crab legs, which can achieve a displacement difference of less than 3% between forward and reverse outputs within 1 s [26]. The above studies indicate that applying the symmetric structures commonly found in most organisms to piezoelectric actuators can significantly improve their bi-directional output performance, thus confirming that the application of bionic technology in the field of piezoelectric actuation is effective and feasible.

The above research results demonstrate that bionic technology is one of the important approaches to enhance the performance of piezoelectric actuators. In this paper, a bi-directional stick-slip piezoelectric actuator with a bionic crab pincer structure is proposed. Based on the symmetric structure of crab pincers, this actuator is designed with two driving feet and adopts an integrated structure to reduce assembly errors. Meanwhile, a backward-motion suppression mechanism and driving electrical signals are designed, and the piezoelectric actuator in this study exhibits high symmetry in bi-directional output performance.

The remaining structure of the paper is as follows. Section 2 introduces the bionic structure and motion principle of the piezoelectric actuator. Section 3 conducts theoretical analysis and calculations on the designed mechanism to obtain the amplification ratio. Section 4 describes the establishment of the experimental platform and the fabrication of the prototype, as well as the testing of the designed piezoelectric actuator. Section 5 summarizes and analyzes the theoretical, simulation, and experimental data to draw conclusions.

## 2. Bionic Structure Design and Motion Principle

### 2.1. Structural Design

**Figure 2:**
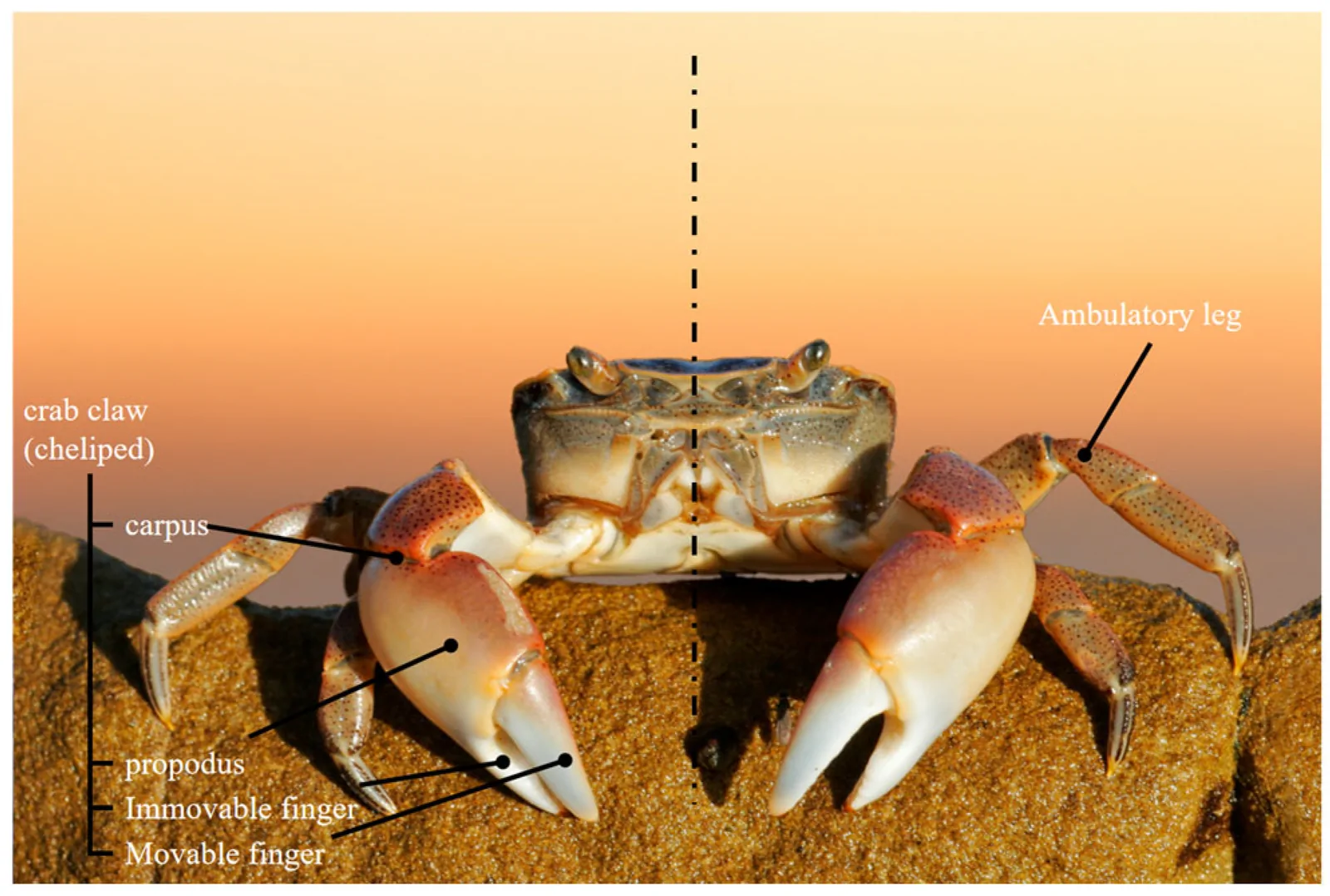
Structure of a Crab.

**Figure 3:**
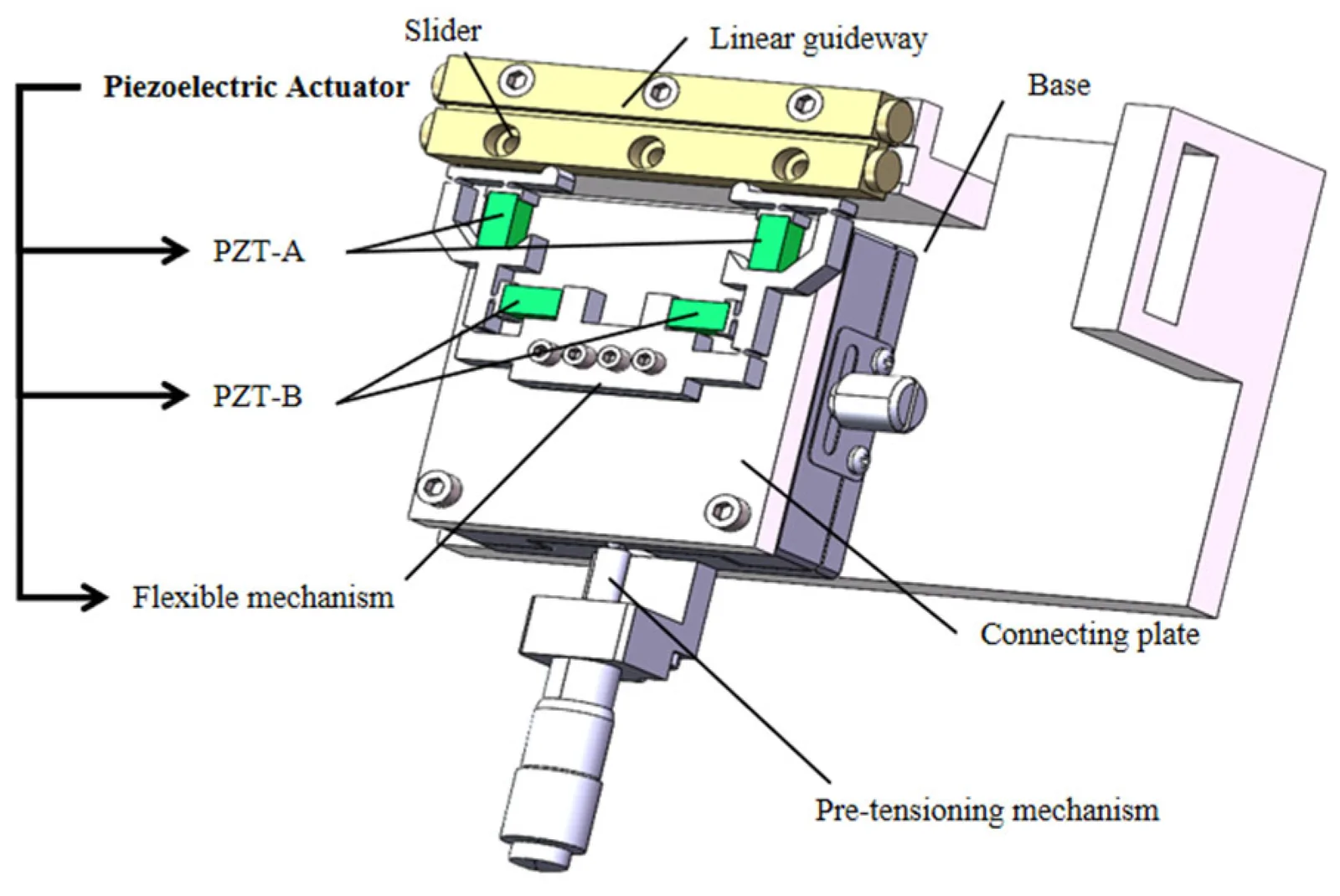
Structure of the Piezoelectric Actuator Positioning Stage.

Crab claws have the characteristic of achieving rapid opening and closing motion through the cooperative movement of joints. It should be noted that crab species show diversity in claw morphology: some species (e.g., fiddler crabs) have asymmetric dimorphic claws, where the major claw is used for courtship display and combat, while the minor claw is for feeding. However, this functional asymmetry does not fit the requirement of bi-directional symmetric output and continuous reciprocating actuation. The bionic prototype of this work is the symmetric chelipeds of swimming crabs (e.g., Portunus trituberculatus), whose left and right claws are highly consistent in size, structure and force output. Such symmetric claws cooperate to perform rapid sand-burrowing and prey-grasping motions, which match the bi-directional, high-frequency stick-slip actuation target of this work. Different from previous bionic crab-claw structures that mainly imitate grasping morphology, this work decouples the sand-burrowing motion of crab claws into two functional degrees of freedom: preload locking (movable finger opening/closing) and tangential drive (carpal joint rotation), which is the core innovation of the proposed actuator. This mechanism of “achieving functional output through rapid motion” is highly consistent with the core operating logic of piezoelectric actuators, which rely on rapid motion to output displacement and suppress backward motion. Therefore, based on the motion characteristics and structure of crab claws (mainly composed of the carpal joint, propodus, movable finger, and fixed finger) as shown in Figure 2, a positioning platform for the bionic stick-slip piezoelectric actuator in this paper is designed, and its structure is shown in Figure 3. The piezoelectric actuator positioning platform consists of a base, a connecting plate, PZT-A (WL5 × 5 × 10/150), PZT-B (WL5 × 5 × 10/150), a flexible driving mechanism (7075AL, 68 mm × 39 mm × 5 mm), a slider (V3-75-10Z), a pre-tensioning mechanism (60 × 60 precision moving platform), and fixing screws. Among them, the connecting plate connects the pre-tensioning mechanism with the designed piezoelectric actuator; the flexible mechanism, fixing screws, PZT-A, and PZT-B together form the stator; and the slider and guideway form the mover.

### 2.2. Motion Principle (TCF)

**Figure 4:**
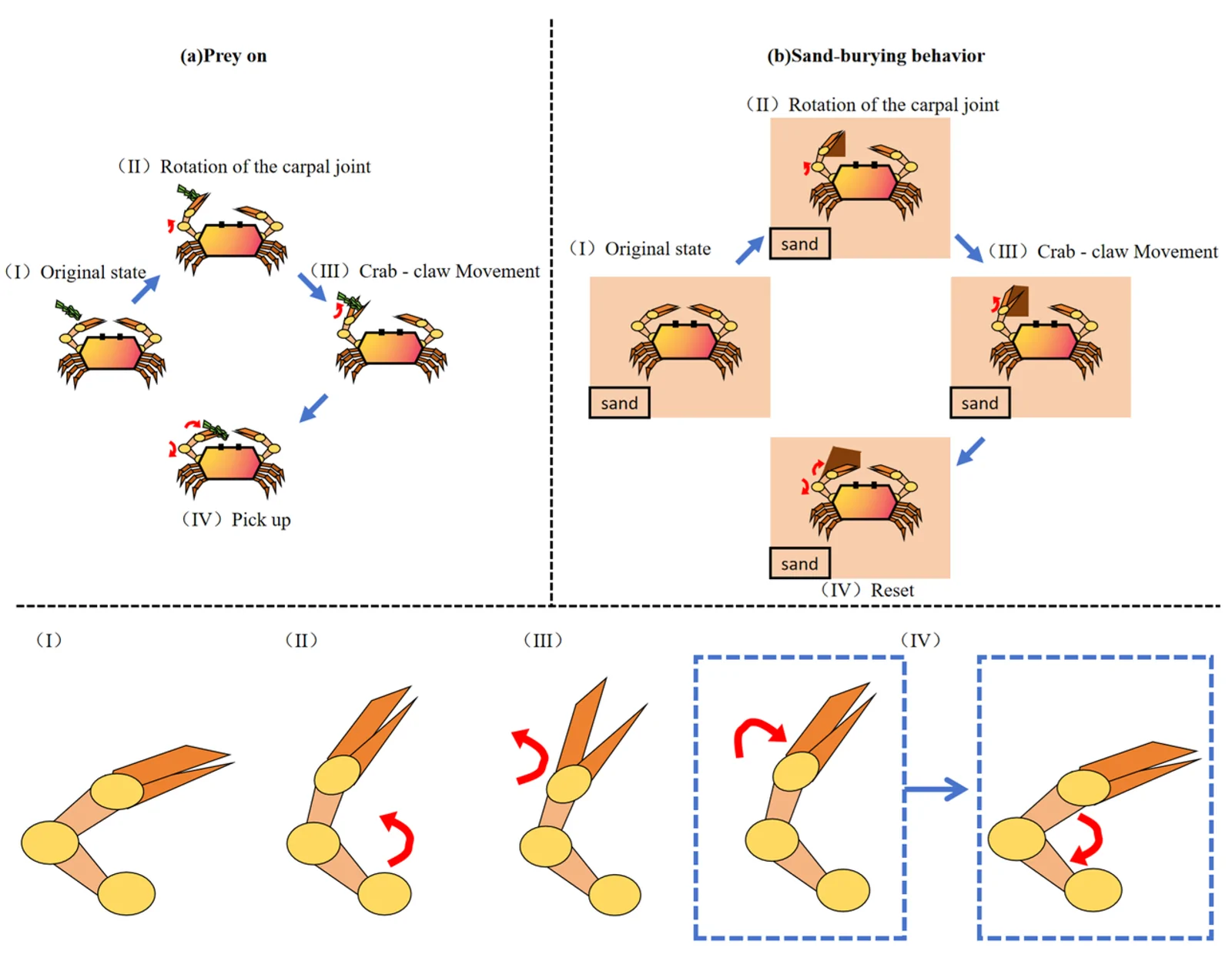
Schematic diagram of crab pincer motion. (**a**) Prey on; (**b**) sand-burying behavior.

**Figure 5:**
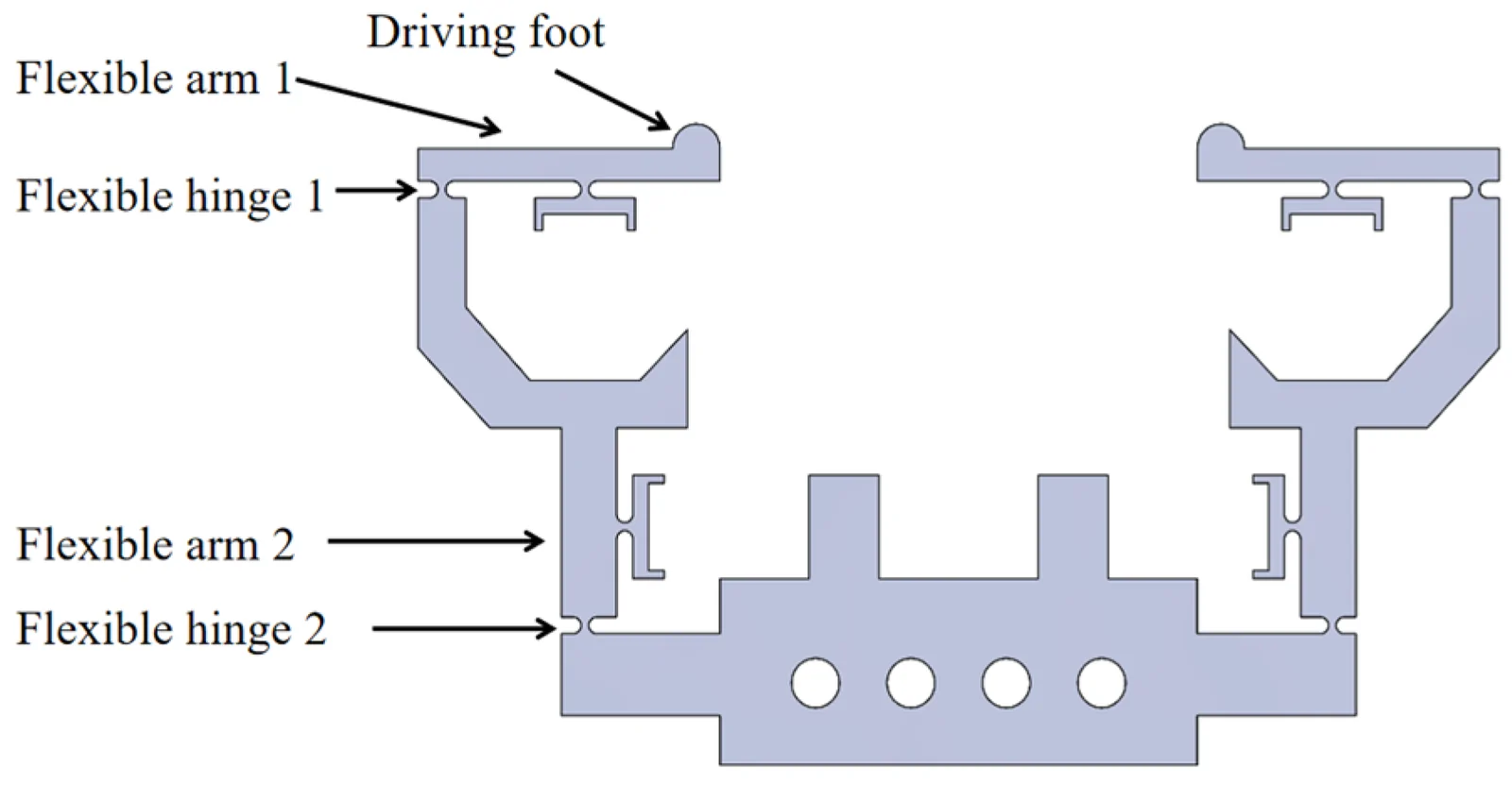
Schematic Diagram of the Flexible Structure.

Crabs mainly control the opening and closing of the movable finger and the rotation of the carpal joint through the muscles in the crab claw to grip objects. Crabs also have the habit of burrowing in sand. When burrowing, crabs will quickly and repeatedly perform the combined motion of opening and closing the crab claw and rotating the carpal joint to push away the surrounding sand and burrow into the sand. The specific motion principle is shown in Figure 4, which clearly shows the opening and closing actions of the crab claw in the two types of motion. Taking the grasping motion and sand-burrowing motion of the crab claw as the basis for designing the working principle of the piezoelectric actuator, we propose the TCF (Total Cancelation Force) actuation mode. In this mode, different voltages are applied to the two piezoelectric ceramics to drive the flexible mechanism and generate telescopic motion within one motion cycle. The specific composition of the flexible mechanism is shown in Figure 5. The telescopic motion of PZT-A drives the rotation of Flexible hinge 1, simulating the opening and closing of the movable finger of the crab claw; the telescopic motion of PZT-B drives the rotation of Flexible hinge 2, simulating the rotational motion of the crab’s carpal joint.

For the selection of the driving electrical signal of the piezoelectric ceramic, the traditional sawtooth wave electrical signal will cause damage to the piezoelectric actuator mechanism, lead to backward motion phenomenon, and generate significant noise that interferes with the working environment [27,28]. However, the trapezoidal wave can not only make the piezoelectric ceramic move quickly through the electrical signals of the rising and falling edges, but also the level part can keep the mechanism in a stable state for a period of time, providing a buffer time during continuous periodic motion, which plays a role in stabilizing the start of the next motion and protecting the mechanism. Therefore, the working waveform of the piezoelectric actuator adopts a trapezoidal wave as the driving electrical signal.

The electrical signal for controlling the drive in this paper first quickly reaches the specified working voltage through a rising edge. Then, the electrical signal is controlled to remain at a high level for a period of time, and then reduced to the initial voltage through a falling edge. After controlling the actuator to complete a single step within one motion cycle, the deformed structure is kept for a period of time to avoid the retraction motion of the stator (piezoelectric actuator) caused by the sudden voltage drop from driving the mover, which is still in the adhesion stage, to generate reverse backward motion and affect the output performance.

**Figure 6:**
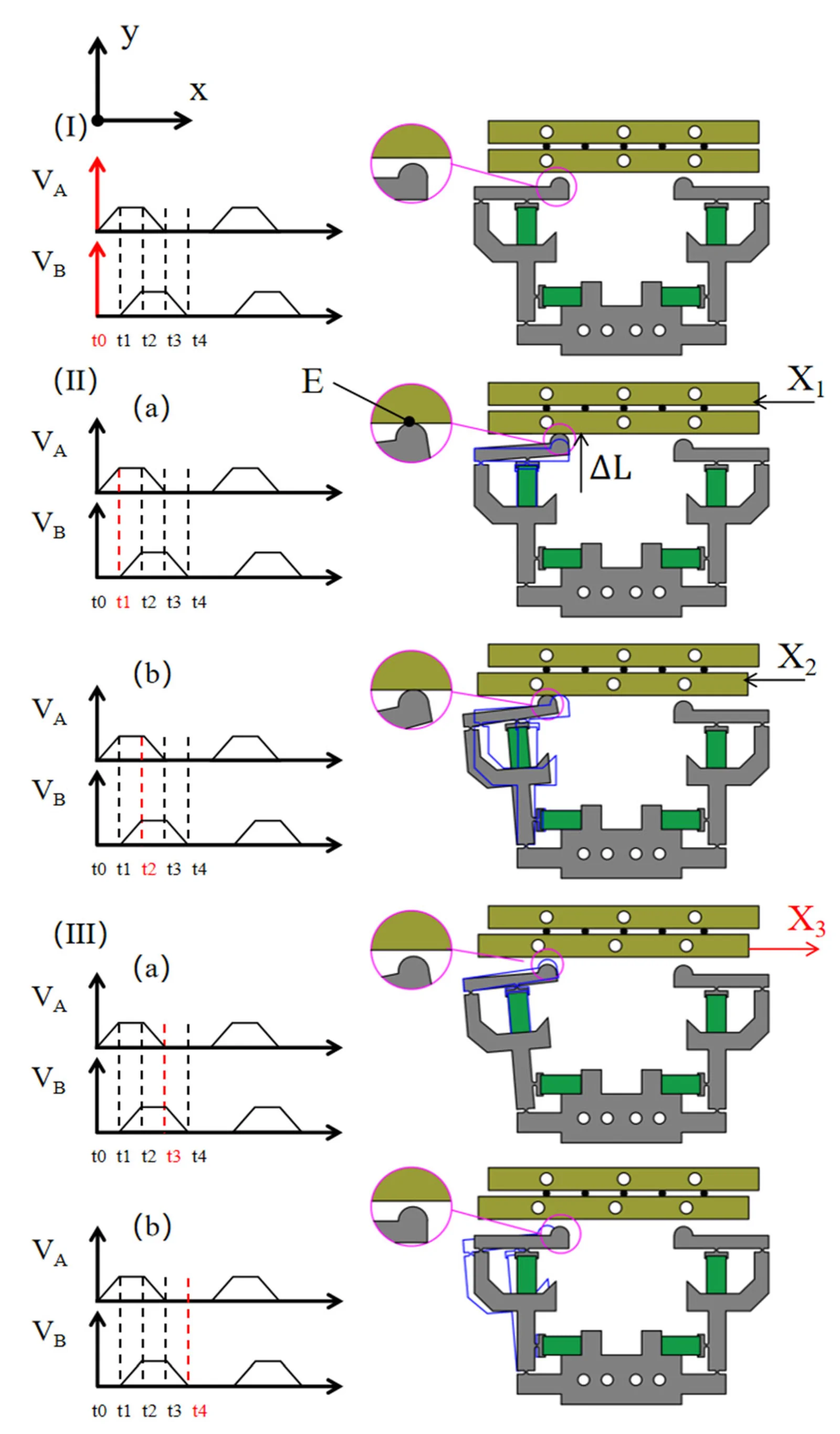
Unidirectional working principle of the piezoelectric actuator. (**I**) Initial stage. (**II**) Stick-slip output stage: (a) stick; (b) slip. (**III**) Retraction and reset stage: (a) retraction; (b) reset.

Based on this, the working process of the designed stick-slip piezoelectric actuator is initially divided into three parts: the initial stage, the stick-slip output stage, and the retraction reset stage. Figure 6 shows the leftward working principle of the left side (same as the rightward working principle of the right side). Under the maximum working voltage of 150 V (PZT-A extends by 8 μm, PZT-B extends by 8 μm), the motion process is as follows (ΔL is the pre-tensioning displacement):

(I) Initial Stage: At the initial moment t_0_, PZT-B is kept stationary, and a rising-edge electrical signal is applied to PZT-A to make it extend.
(II) Stick-Slip Output Stage:
  (a) Stick: During t_0_–t_1_, the extension motion of PZT-A drives Flexible hinge 1 to rotate counterclockwise, causing the upper part of the structure to generate an upward displacement. At moment t_1_, PZT-A reaches the maximum extension, making the Driving foot generate a motion in the positive *y*-axis direction to produce a locking displacement ΔL. At this time, the driving foot contacts and adheres to the slider (contact point E), and outputs a displacement X_1_ in the negative x-direction. Meanwhile, a rising-edge electrical signal is applied to PZT-B.
  (b) Slip: During t_1_–t_2_, the voltage signal of PZT-A is always kept at a high level to fix the position of the driving foot and the slider in the y-direction. PZT-B generates an extension through the rising-edge electrical signal to drive Flexible hinge 2 to rotate clockwise, so that the driving foot of the flexible mechanism drives the slider to generate an output displacement X_2_ in the negative x-direction, and reaches the maximum single-step output displacement (X_1_ + X_2_) at t_2_.
(III) Retraction and Reset Stage:
  (a) Retraction: During t_2_–t_3_, the high-level electrical signal of PZT-B is maintained to fix the position of the driving foot and the slider in the x-direction (contact state). PZT-A generates a contraction through the falling-edge electrical signal to drive Flexible hinge 1 to rotate clockwise, moving the Driving foot in the negative *y*-axis direction. At t_3_, PZT-A is completely contracted to its initial length (ΔL_1_ is 0).
  (b) Reset: During t_3_–t_4_, a low-level electrical signal is applied to PZT-A to keep it in the initial state, and a falling-edge electrical signal is applied to PZT-B to make it contract. At t_4_, it contracts to its initial length. The contraction process drives Flexible hinge 2 to rotate clockwise, moving the driving foot to the initial state in the negative *x*-axis direction and generating a small backward displacement X_3_. During the motion process, the final output displacement D of a single motion cycle is:

*D* = *X*_1_ + *X*_2_ − *X*_3_ (1)

The coordination mechanism of the two PZTs and the autonomous backward motion suppression principle are explained as follows:

Initial stage (t_0_–t_1_): PZT-A extends first to push the driving foot against the slider, building up normal preload force. This converts the contact interface from a sliding state to a static friction state, completing “autonomous locking” by mechanical structure without additional feedback control.

Stick-slip output stage (t_1_–t_2_): PZT-B extends while PZT-A maintains high voltage to keep locking. The static friction force drives the slider to move forward together with the driving foot, with no relative slip.

Retraction stage (t_2_–t_3_): PZT-A contracts first to release the normal preload, switching the interface from static friction to kinetic friction. At this time, PZT-B still maintains extension to fix the x-direction position of the driving foot.

Reset stage (t_3_–t_4_): PZT-B contracts to drive the driving foot back to the initial position. Since the interface is in a kinetic friction state with low friction force, the reverse dragging effect on the slider is greatly weakened, thus suppressing backward motion.

The term “autonomous” means that the preload locking and friction state switching are realized by the structural deformation of the flexible hinge itself, which only depends on the fixed timing of the two-channel driving signals, and does not require an additional force sensor or closed-loop control system.

### 2.3. Theoretical Calculation

To obtain and verify the specific amplification ratio of the flexible bionic mechanism, the commonly used methods are the pseudo-rigid-body method and the compliance matrix method. Although the pseudo-rigid-body method can assume that the elastic deformation of the flexible mechanism of the actuator is concentrated on the flexible hinges (flexible beams) and is pure bending deformation, thus reducing the calculation difficulty, it leads to cumulative calculation errors in the transmission of the model structure, making it difficult to accurately describe the elastic deformation of the flexible hinge mechanism [29]. In contrast, the compliance matrix method supports coupled deformation of rigid bodies with multiple degrees of freedom and has small calculation errors, so it is adopted to verify the driving amplification ratio of the flexible hinge mechanism [30].

**Figure 7:**
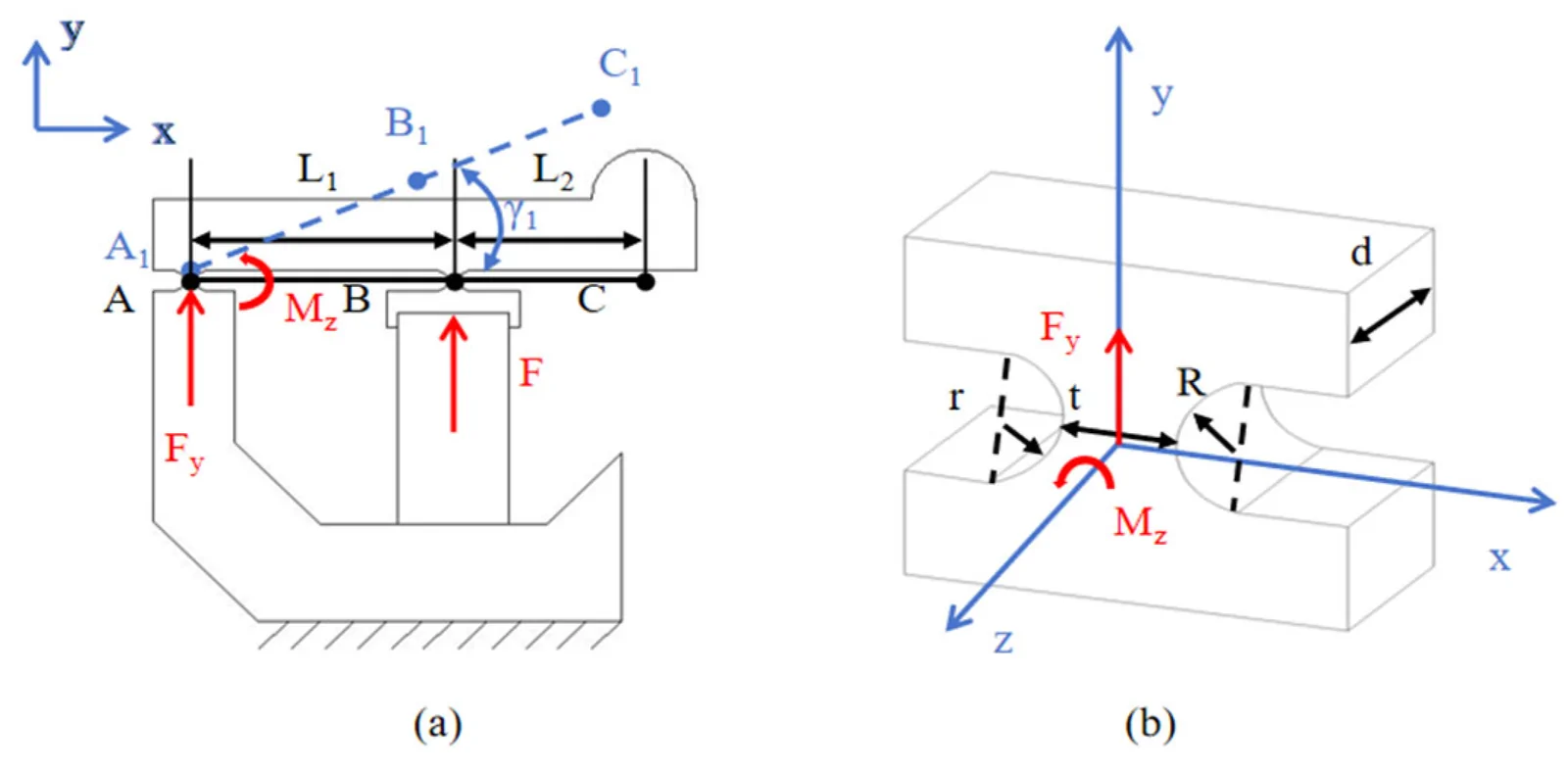
Theoretical model of the preload mechanism. (**a**) Deformation diagram of the preload mechanism; (**b**) flexible hinge model.

Since the piezoelectric actuator works with the designed preload mechanism moving first to suppress the reverse motion of the driving mechanism, the displacement amplification ratio of PZT-A is calculated first. The principle model is shown in Figure 7. The input force F_1_ is generated by the extension of PZT-A; the center position of Flexible hinge 1 is marked as point A with a moment M_z_, the length from point A to the output point B of PZT-A is L_1_, and the length from point B to the driving foot point C is denoted as L_2_. At this time, without considering the driving mechanism, the bottom beam of PZT-A is regarded as a fixed constraint. Then the angular deformation γ_1_ of Flexible hinge 1 around the *z*-axis can be expressed as:

(2) $$\gamma_{1}=\frac{d\theta_{z}}{dF_{x}}\times F_{x}+\frac{d\theta_{z}}{dM_{z}}\times M_{z}$$

Since the deformation of Flexible hinge 1 mainly plays a preloading role and has little effect on the output displacement direction x of the mechanism, the y-direction deformation Dy of Flexible hinge 1 can be expressed as:

(3) $$D_{y}=\frac{dy}{dF_{y}}$$

The relationship between the output displacement L_outA_ and the input displacement L_inA_ of Flexible hinge 1 can be obtained as:

*L*_*outA*_ = (*L*_1_ + *L*_2_) × *γ*_1_ + *D*_*y*_ (4)

*L*_*inA*_ = *L*_1_ × *γ*_1_ + *D*_*y*_ (5)

where L_1_ is 9 mm and L_2_ is 7 mm. The amplification ratio n_1_ can be expressed as:

(6) $$n_{1}=\frac{L_{\mathrm{outA}}}{L_{\mathrm{inA}}}$$

The displacement amplification ratio n_1_ of the preload mechanism is 1.778.

**Figure 8:**
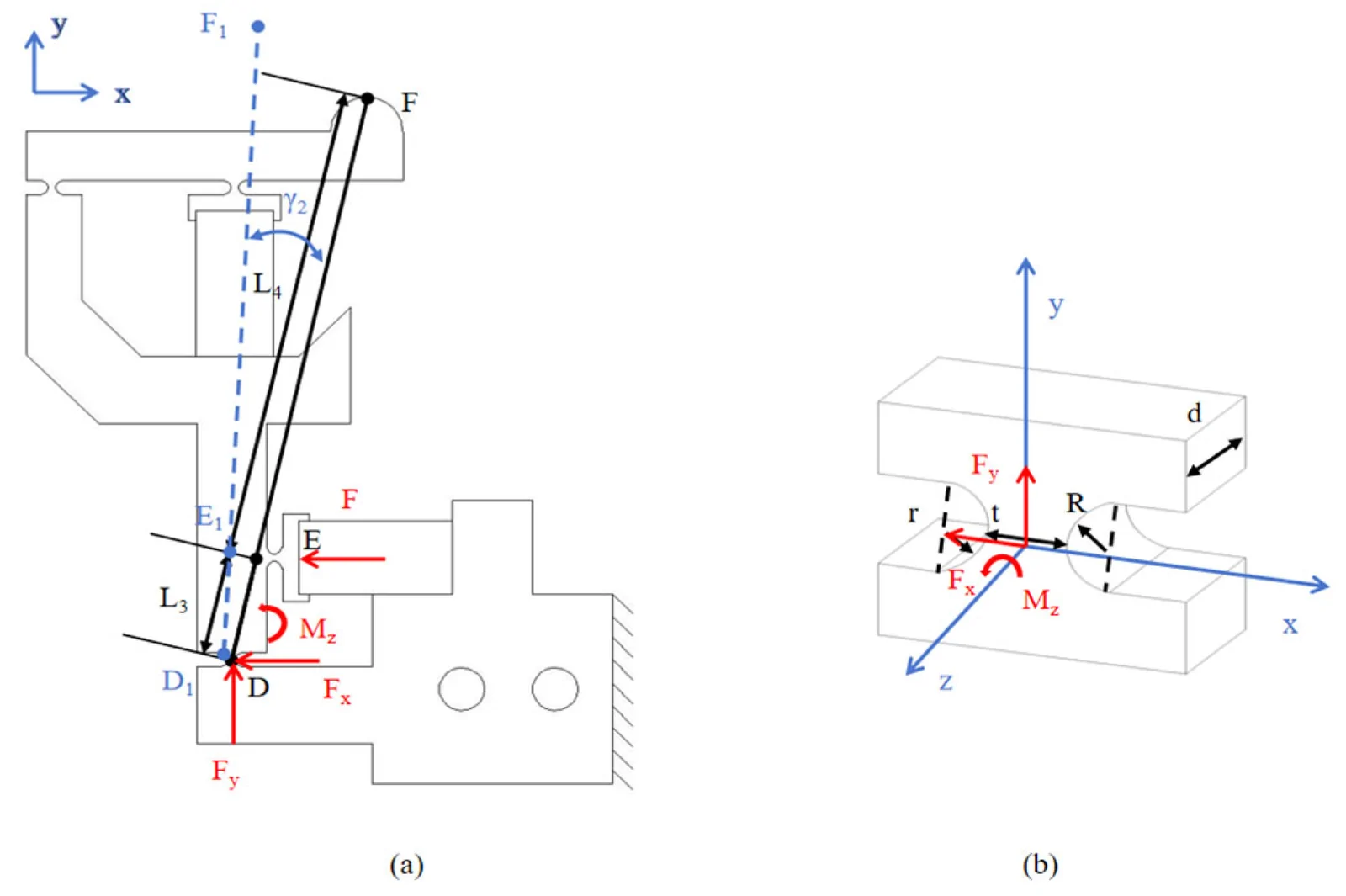
Theoretical model of the output mechanism. (**a**) Deformation diagram of the output mechanism; (**b**) flexible hinge model.

The driving mechanism mainly transmits the input displacement of PZT-B through Flexible hinge 2. Therefore, when calculating the amplification ratio of the driving mechanism, only the driving displacement is calculated without considering the deformation of the preload mechanism. Its principle model is shown in Figure 8. According to the compliance matrix, the angular deformation γ_2_ of Flexible hinge 2 can also be expressed by Equation (2). The x-direction deformation D_x_ of Flexible hinge 2 can be expressed as:

(7) $$D_{x}=\frac{dx}{dF_{x}}\times F_{x}+\frac{dx}{dM_{z}}\times M_{z}$$

The y-direction deformation of Flexible hinge 2 is expressed by Equation (3). The relationship between the output displacement L_outB_ and the input displacement L_inB_ of Flexible hinge 2 can be obtained as:

*L*_*outB*_ = (*L*_1_ + *L*_2_) × *γ*_2_ + *D* (8)

*L*_*inB*_ = *L*_2_ × *γ*_2_ + *D* (9)

where D is the total displacement of Flexible hinge 2, the length L_3_ from point D to point E is 6.15 mm, and the length L_4_ from point E to point F is 24.15 mm. The final amplification ratio of the output mechanism is expressed as:

(10) $$n_{2}=\frac{L_{\mathrm{outB}}}{L_{\mathrm{inB}}}$$

The displacement amplification ratio n_2_ of the output mechanism is 4.927.

The detailed calculation of angular deformation displacement can be obtained from the formula proposed by Shen [31], where r is equal to R:

(11) $$\theta =\int_{R-r}^{R+r}\frac{12M}{Eb{\left[R+r+t-\sqrt{R^{2}-{\left(R-x\right)}^{2}}-\sqrt{r^{2}-{\left(R-x\right)}^{2}}\right]}^{3}}\mathrm{dx}$$

## 3. Mechanical Performance Analysis

### 3.1. Static Analysis

To verify the accuracy of the theoretical analysis and determine the characteristics of the actuator, the finite element analysis method is adopted in this paper to conduct static analysis on the designed piezoelectric actuator structure. The actuator is placed in the simulated displacement physical field, and the material of the actuator is set to AL7075. The elastic modulus, material density, and Poisson’s ratio are 71.7 GPa, 2810 kg/m^3^, and 0.33, respectively, as shown in Table 1.

**Figure 9:**
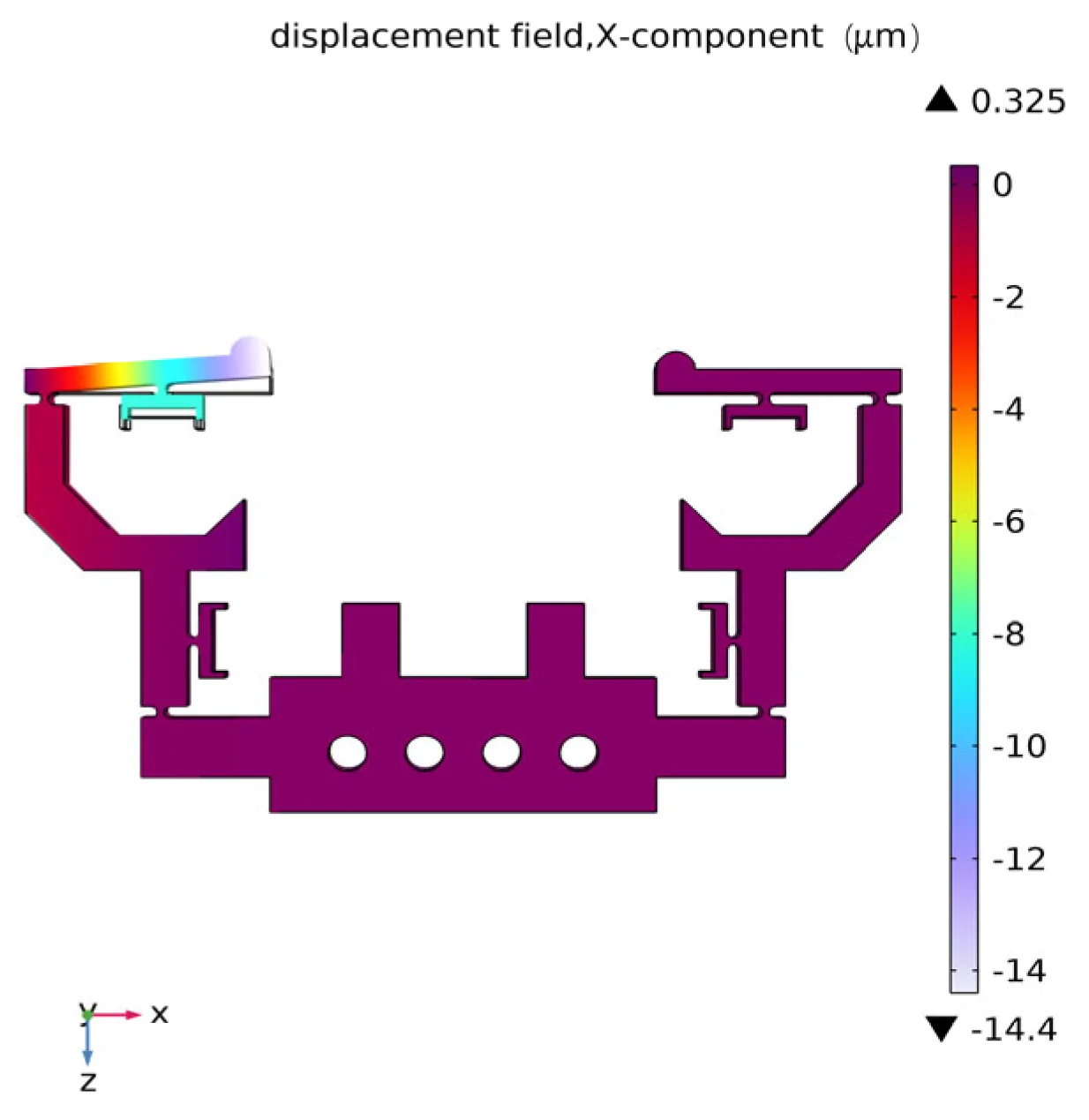
Static analysis results under the operation of PZT−A.

**Figure 10:**
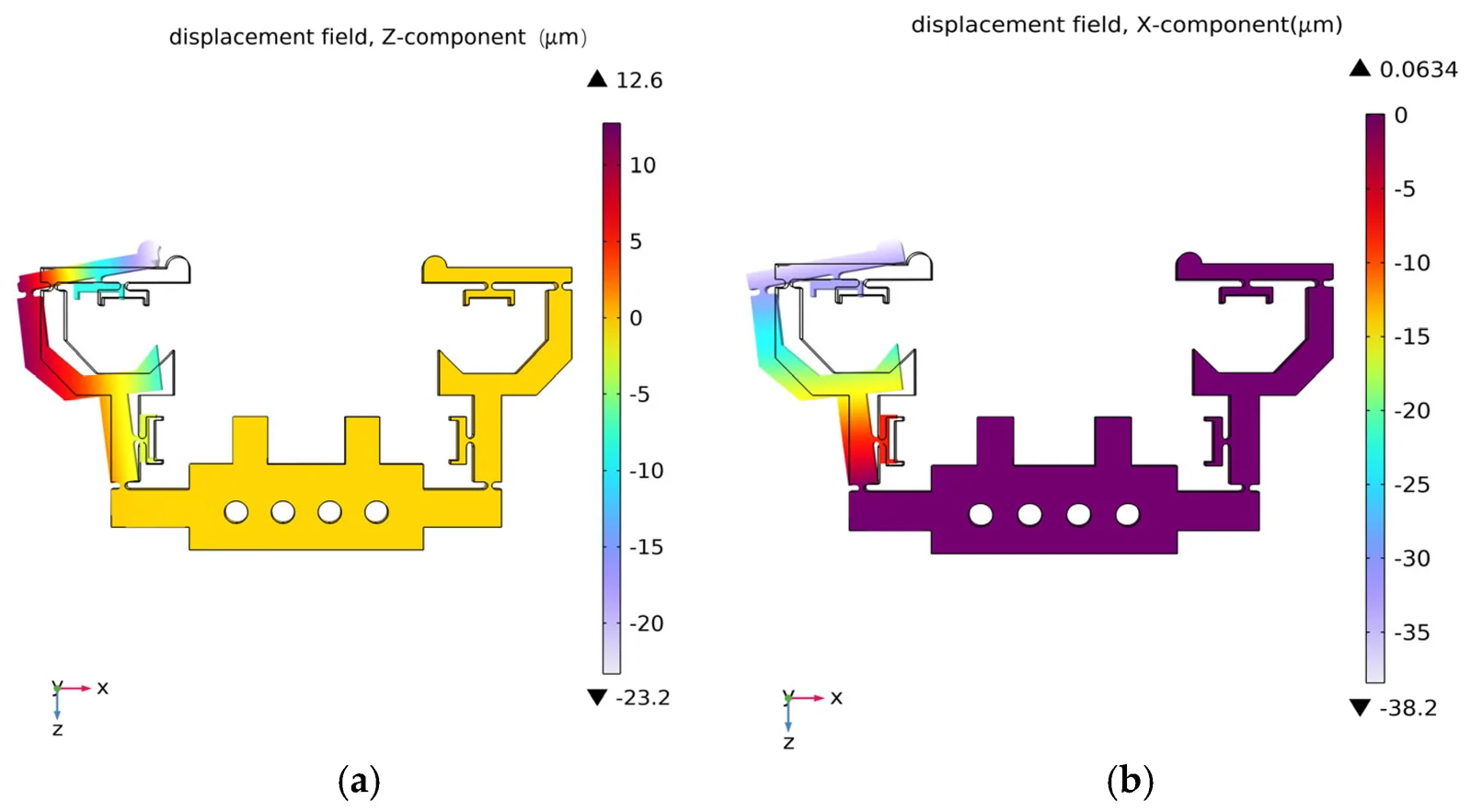
Static analysis results under the operation of PZT−A and PZT−B. (**a**) Pre−tensioning direction; (**b**) output direction.

Fixed constraints are applied to the four screw holes. Finite element simulation is performed with a mesh containing 70,808 elements and an average element quality of 0.65. The maximum extension of PZT-A and PZT-B under a 150 V voltage is regarded as the displacement, and a displacement of 8 μm is sequentially applied to the action mechanism at the action head of the piezoelectric ceramic. When PZT-A works alone, the driving foot after applying displacement will generate a main displacement of 14.4 μm in the pre-tensioning direction, and the result is shown in Figure 9. When both PZT-A and PZT-B work simultaneously, the driving foot after applying displacement will generate displacements in the vertical and horizontal directions. Figure 10 shows a maximum horizontal output displacement of 38.2 μm and a maximum vertical displacement of 23.2 μm. The main displacement errors obtained by the theoretical calculation and the finite element analysis method are shown in Table 2. When PZT-A works alone, the error between the theoretical calculation result and the finite element analysis result is 1.39%. When both PZT-A and PZT-B work simultaneously, the error between the theoretical calculation result and the simulation result is 3.66%. The slightly larger error under dual-PZT actuation mainly comes from two aspects:

1. Deformation coupling effect: The theoretical calculation based on the compliance matrix method assumes that the deformations of flexible hinge 1 (preload mechanism) and flexible hinge 2 (drive mechanism) are independent of each other. However, in finite element simulation, the extension of PZT-B will induce additional micro-deformation of flexible hinge 1, and vice versa. This coupling effect is not considered in the theoretical model, resulting in a larger deviation.
2. Deformation mode complexity: The deformation of PZT-A alone is dominated by y-direction preload displacement with a simple bending mode, so the theoretical assumption fits the actual situation better. When both PZTs work simultaneously, the driving foot undergoes combined displacement in x and y directions, and the theoretical model simplifies secondary shear deformation, leading to a slight increase in error.

Even so, the maximum error of 3.66% is still within the acceptable range of engineering calculation (<5%), which verifies the accuracy and reliability of the theoretical model.

**Figure 11:**
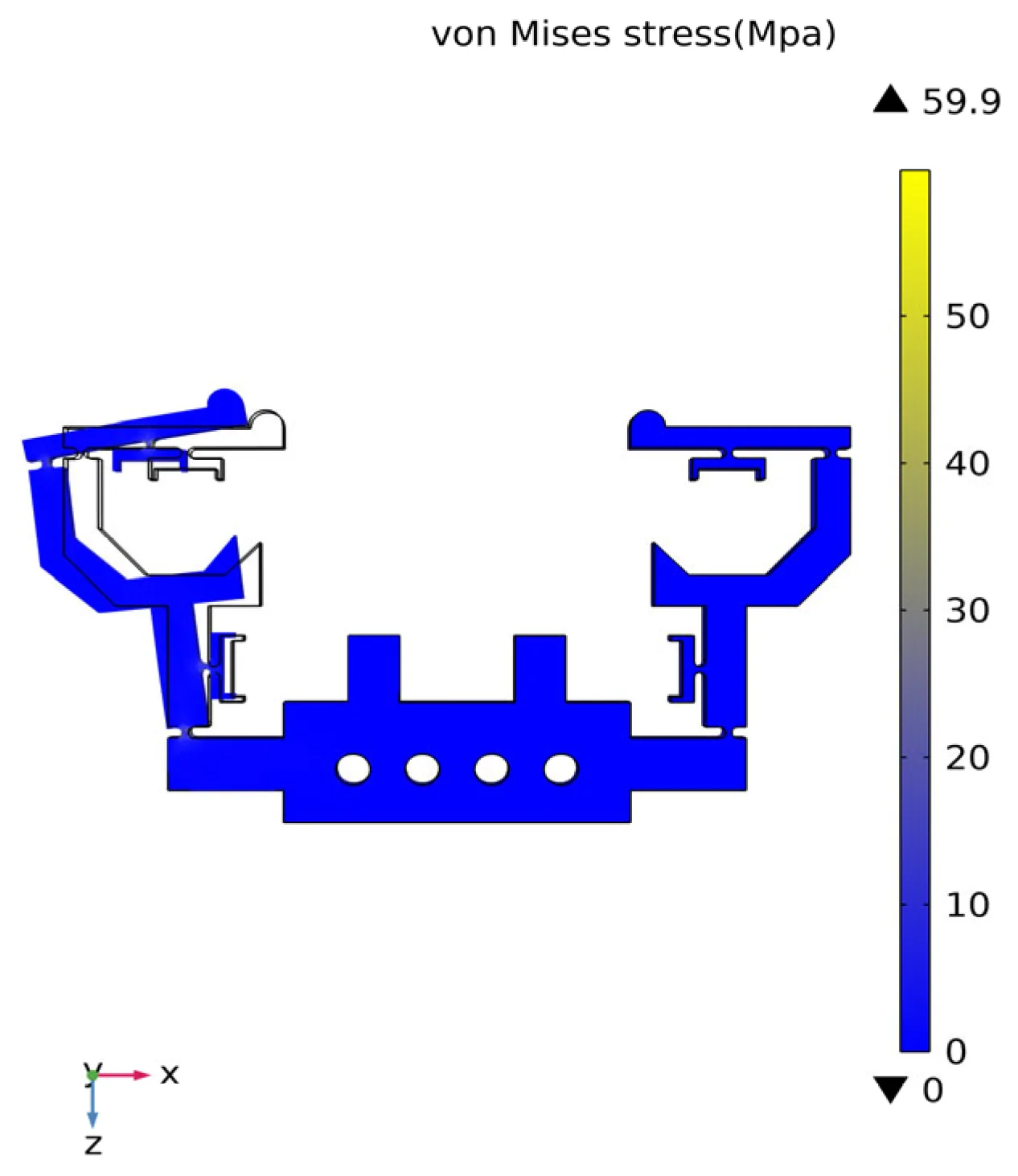
Stress analysis.

To test the strength of the actuator, the stress analysis of the static mechanics in the finite element simulation is further conducted, and it is found that the maximum stress on the piezoelectric actuator during operation is at the flexible hinge. As shown in Figure 11, the results indicate that the maximum stress on the entire mechanism is 59.9 MPa, which is much lower than the yield strength of 500 MPa of the actuator material (AL7075). This verifies that the designed mechanism will not break when the piezoelectric ceramic works at the maximum voltage (150 V) and has sufficient working strength.

### 3.2. Modal Analysis

Modal analysis is essential for stick-slip piezoelectric actuator design for two core reasons: first, the actuator is driven by periodic electrical signals; if the operating frequency approaches the natural frequency of the structure, resonance will occur, causing unstable output, increased stress concentration, accelerated PZT fatigue, and excessive temperature rise. Second, modal analysis quantitatively obtains the natural frequency distribution and vibration modes, guiding the selection of the operating frequency range to avoid resonance damage.

**Figure 12:**
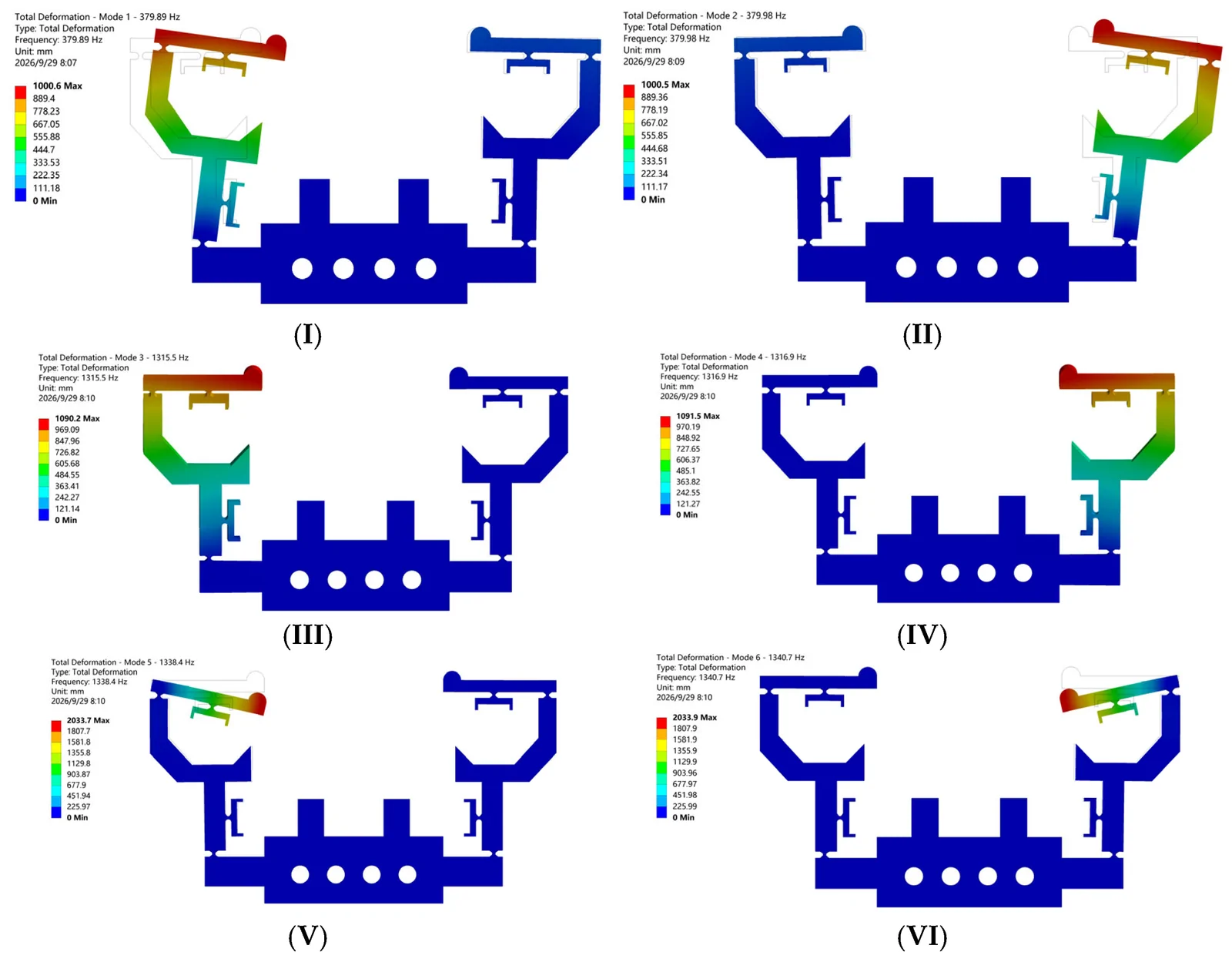
Modal analysis results of the actuator: (**I**) First-order mode; (**II**) second-order mode; (**III**) third-order mode; (**IV**) fourth-order mode; (**V**) fifth-order mode; (**VI**) sixth-order mode.

To protect the flexible mechanism and the piezoelectric ceramic, modal analysis of the mechanism is conducted via the finite element method. The simulation mesh has an average element quality of 0.84, which guarantees the reliability of the calculation results. The six-order natural frequencies of the mechanism are obtained and listed in Table 3. The mode shapes corresponding to the first-order to sixth-order natural frequencies are presented in Figure 12. The results indicate that these frequencies should be avoided during experiments and operations to prevent resonance of the mechanism, which would cause increased energy consumption, local temperature rise and potential damage to the mechanism.

The first-order natural frequency of the structure is 379.89 Hz, while the maximum operating frequency in this work is 100 Hz, which accounts for only 26.3% of the first-order resonance frequency. This provides a sufficient safety margin and ensures that the actuator operates in the quasi-static region without resonance risk.

The similar shapes of adjacent modes arise from the high left-right symmetry of the designed structure: the first-order and second-order modes form a pair of symmetric and antisymmetric in-plane bending modes; the third-order and fourth-order modes constitute a pair of torsional modes; the fifth-order and sixth-order modes make up a pair of out-of-plane bending modes. The natural frequencies of each pair are very close in value, and their mode shapes are similar but with opposite vibration directions, which is a typical characteristic of highly symmetric structures. The closeness between the first-order and second-order, third-order and fourth-order, as well as fifth-order and sixth-order natural frequencies further confirms that the designed flexible structure has a high degree of left-right symmetry.

## 4. Experimental Testing

### 4.1. Experimental System

**Figure 13:**
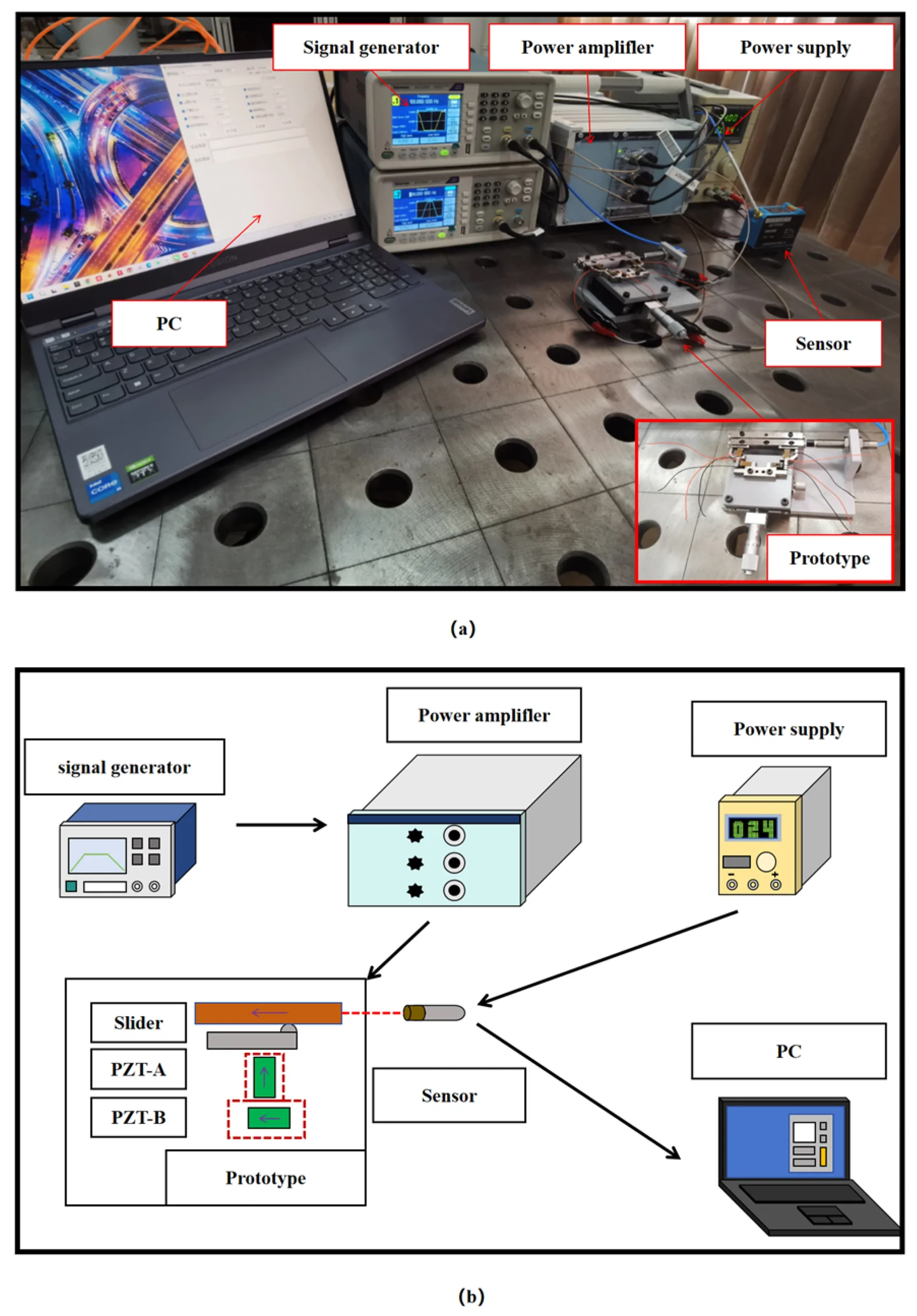
Experimental System. (**a**) Experimental platform (**b**) Experimental principle.

To further verify the reliability of the performance of the designed actuator, a prototype is fabricated on a 7075AL material block using the wire-cutting process. The experimental materials used include four PZT-5H (PZT-A, PZT-B) piezoelectric ceramics (Guangzhou Future Material Technology Co., Ltd., China), a slider, a precision displacement platform, and a 3D-printed base with holes. The experimental equipment includes a signal generator (AFG1000, Tektronix, USA), a power amplifier (E03 A3, Harbin Core Tomorrow Science & Technology Co., Ltd., China), a 24 V power supply (QJ3005N, Ningbo Jiuyuan Electronics Co., Ltd., China), an eddy current displacement sensor (SACWY002, Wuxi Shiao Technology Co., Ltd., China), and a computer. After assembling the experimental materials, they are placed on the built anti-vibration experimental platform, as shown in Figure 13a.

The operating principle of the experimental system is shown in Figure 13b. The 24 V power supply is used to power the eddy current displacement sensor. First, the experimenter adjusts the trapezoidal wave through the signal generator, couples the waveforms used by the two PZT piezoelectric ceramics, and transmits the emitted waveform electrical signal to the signal amplifier to amplify the voltage. The amplified electrical signal waveform is then applied to the PZT-A and PZT-B piezoelectric ceramics in the prototype, respectively. After receiving the amplified voltage, the piezoelectric ceramics generate regular telescopic motion, which drives the driving foot of the piezoelectric actuator to move. Finally, the movement of the driving foot drives the slider to move, and the eddy current sensor transmits the detected displacement information of the slider movement to the computer for data collection.

### 4.2. Experimental Results

The performance of the actuator is tested using the experimental system, and the maximum driving voltage U of PZT-A and PZT-B is 150 V. To highlight the effectiveness of the designed backward motion suppression mechanism, an experimental verification of the backward motion suppression structure of the actuator is conducted. We set the Non-Preload Force (NPF) motion mode as the control group. In the NPF mode, PZT-B is driven by exactly the same electrical signal as it uses in the TCF mode, while PZT-A is not energized and performs no preload locking action. Its performance is compared with that of the bionic crab claw-inspired TCF motion mode, where PZT-A and PZT-B operate cooperatively to realize preload locking and backward motion suppression.

**Figure 14:**
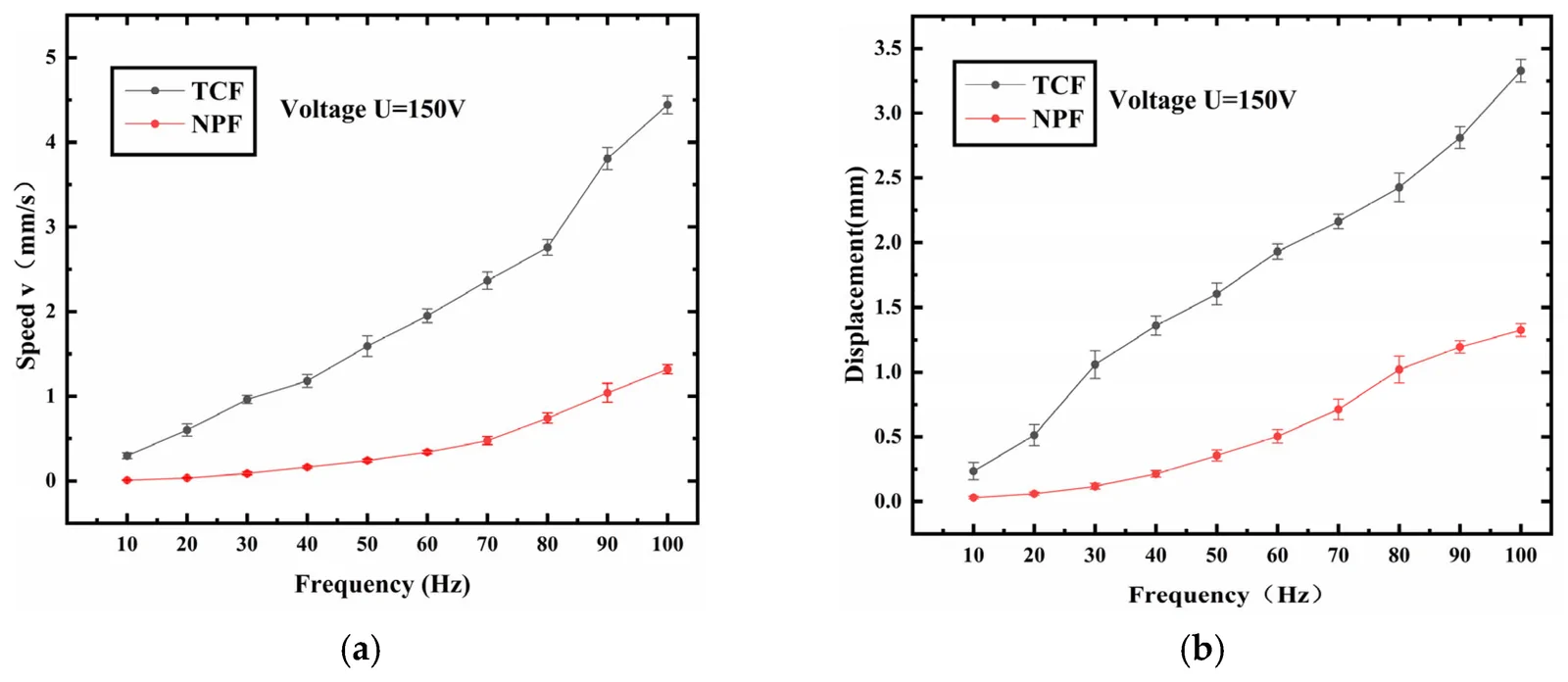
Comparison of TCF and NPF motion modes. (**a**) Relationship between frequency and speed (**b**) Relationship between frequency and displacement.

The experimental results are shown in Figure 14, in which Figure 14a depicts the output speed versus driving frequency, and Figure 14b illustrates the output displacement versus driving frequency. All measurements are carried out under the maximum working voltage of 150 V, with the driving frequency ranging from 10 Hz to 100 Hz. All data are expressed as mean ± standard deviation based on five independent repeated tests. The relative standard deviation is maintained at 3–8% for the TCF mode and 5–10% for the NPF mode in the 40–100 Hz middle and high frequency bands, demonstrating favorable experimental repeatability. The slightly increased relative deviation at low-frequency points is an inherent characteristic of precision stick-slip driving systems, since the micro-fluctuations of friction force and measurement resolution account for a larger proportion when the output amplitude is small.

The output speed and displacement of both modes show an approximately linear growth trend with the increase in driving frequency, which is consistent with the basic operation law of stick-slip piezoelectric actuators, indicating that the actuator works stably without step-out in the 10–100 Hz frequency band. The TCF mode always presents better output performance than the NPF mode in the whole test frequency range. At the low frequency of 10 Hz, the output of the NPF mode is nearly zero due to the prominent backward motion, while the TCF mode still maintains stable and effective output. In addition, as the driving frequency increases gradually, the output performance of the TCF mode shows a growing advantage over the NPF mode in both output speed and output displacement, reaching a maximum output speed of 4.58 mm/s at 100 Hz. On the one hand, the PZT piezoelectric ceramic under 150 V provides sufficient driving force to drive the actuator to move; on the other hand, the designed backward motion suppression mechanism can effectively suppress the backward motion of the actuator in both high-frequency and low-frequency working environments, improving the displacement output rate. Thus, the reliability of the backward motion suppression mechanism is verified.

**Figure 15:**
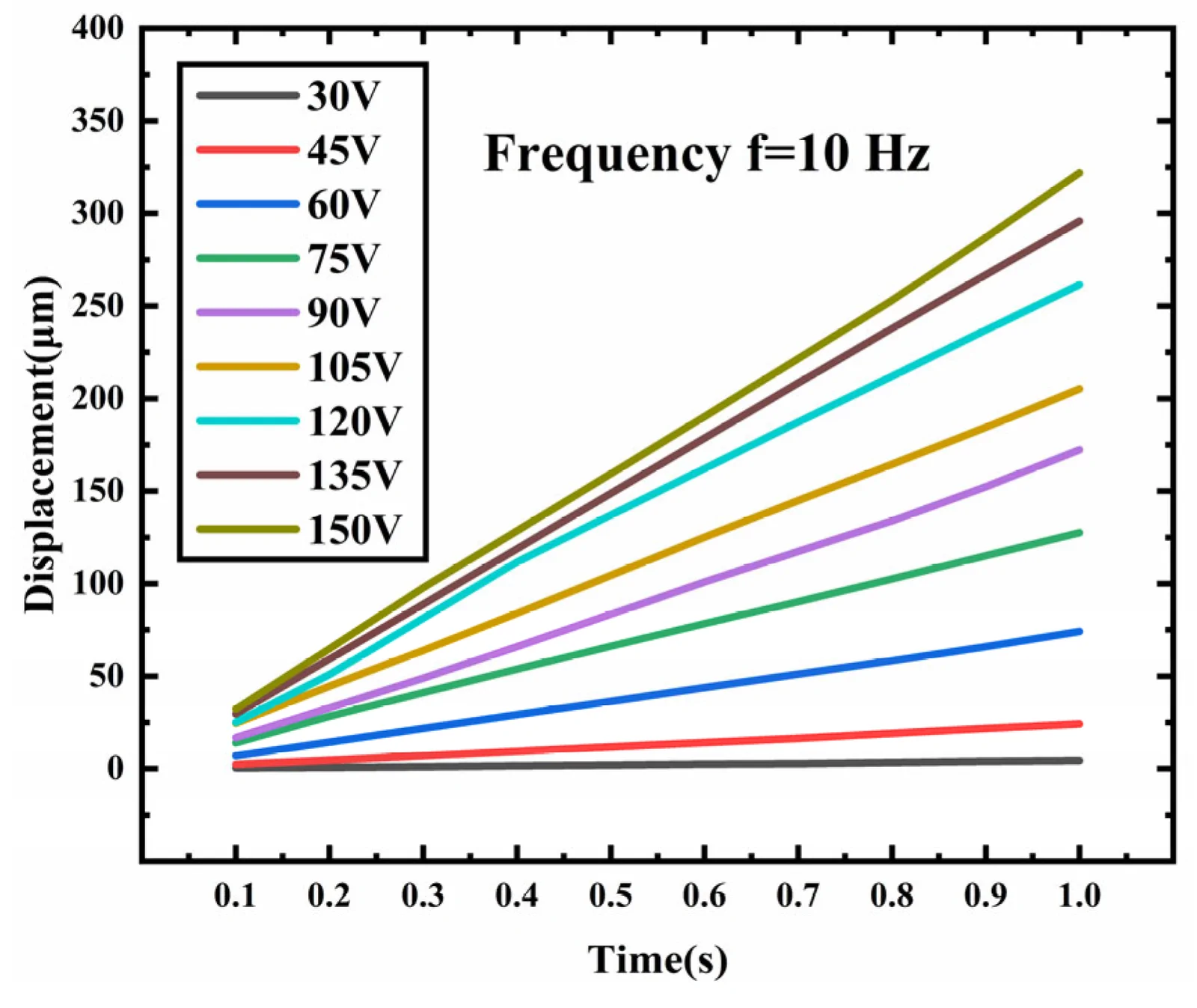
Relationship between output displacement and voltage in TCF motion mode.

To obtain the actual driving amplification ratio of the flexible hinge mechanism, the output displacement of the flexible hinge mechanism in the driving direction is tested under a driving voltage of 150 V and 10 Hz. As shown in the experimental result in Figure 15, the input displacement of the piezoelectric ceramic under this driving voltage is 8 μm, and the single-step output displacement is 37.9 μm. Thus, the actual driving amplification ratio of the flexible hinge mechanism is calculated to be 4.738, as shown in Table 4. Compared with the amplification ratio analysis results of Calculation and FEM, the maximum error is 3.99%, which is due to unavoidable processing errors, resulting in the actual amplification ratio being lower than the theoretical calculation and simulation analysis results. Overall, the experimental results agree well with theoretical and simulation results: the single-step output displacement also shows the same consistent trend (39.6 μm in theory, 38.2 μm in simulation, and 37.9 μm in experiment). The deviation mainly comes from wire-cut thickness tolerance of flexible hinges, assembly gaps, the difference between actual and nominal PZT displacement, and friction energy loss at the contact interface.

Further experimental verification is conducted on the output performance of the TCF motion mode. To obtain the relationship between the output displacement and the working voltage, the working environment is set to a fixed low frequency of 10 Hz, and the voltage is changed as shown in the experimental result in Figure 15. When the voltage is in the range of 60 V to 105 V, the output performance of the piezoelectric actuator is significantly improved compared with that when the working voltage is lower than 60 V. This is because under the initial pre-tensioning displacement (0 μm), the working voltage causes the piezoelectric ceramic to extend, driving the driving foot to cling to the slider and providing sufficient preload to effectively suppress the reverse motion, enabling the actuator to output displacement well. However, when the voltage is in the range of 105 V to 150 V, although the output displacement still increases with the increase in voltage, the line segment at this time is denser compared with that at the working voltage of 105 V, indicating that the growth of displacement output becomes smaller. This is because the preload between the slider and the driving foot increases with the increase in the extension of the PZT piezoelectric ceramic, which affects the output performance to a certain extent. Therefore, the optimal working voltage range is 60 V to 105 V.

**Figure 16:**
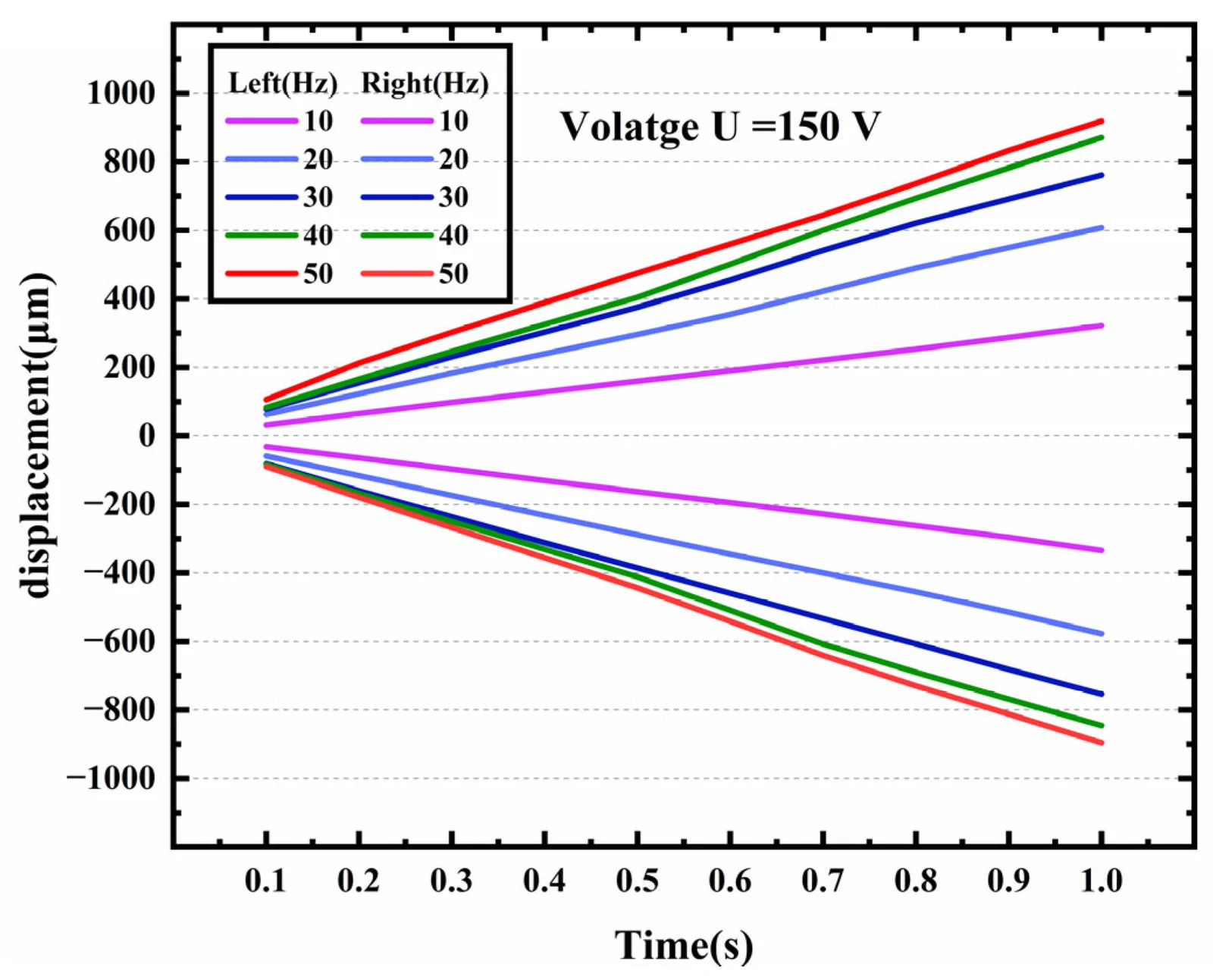
Comparison of forward and reverse output displacements of the actuator at different frequencies.

Due to the high structural symmetry of the actuator, it can move in two opposite directions. The bi-directional output performance of the piezoelectric actuator is tested as shown in Figure 16. It is found that under a voltage drive of 150 V and 50 Hz within 1 s, the forward and reverse motion output displacements are 918.96 μm and 896.03 μm, respectively, and the output displacement error between the two directions is only 2.49%. Among them, the displacement error refers to the ratio of the absolute value of the displacement difference between the reverse motion and the forward motion to the forward motion displacement. The error results under other working conditions are shown in Table 5. The maximum output displacement error between the forward and reverse directions is only 5.02%, which indicates that the designed actuator also has a high degree of bi-directional symmetry in performance. It can also be seen from the experimental results that the displacement curve at 30–50 Hz in the Figure becomes increasingly dense. This is because at a voltage of 150 V, along with the increase in the working frequency, the output displacement within 1 s increases gradually, but the higher the frequency, the slower the increasing trend of displacement becomes, and it gradually tends to be stable.

**Figure 17:**
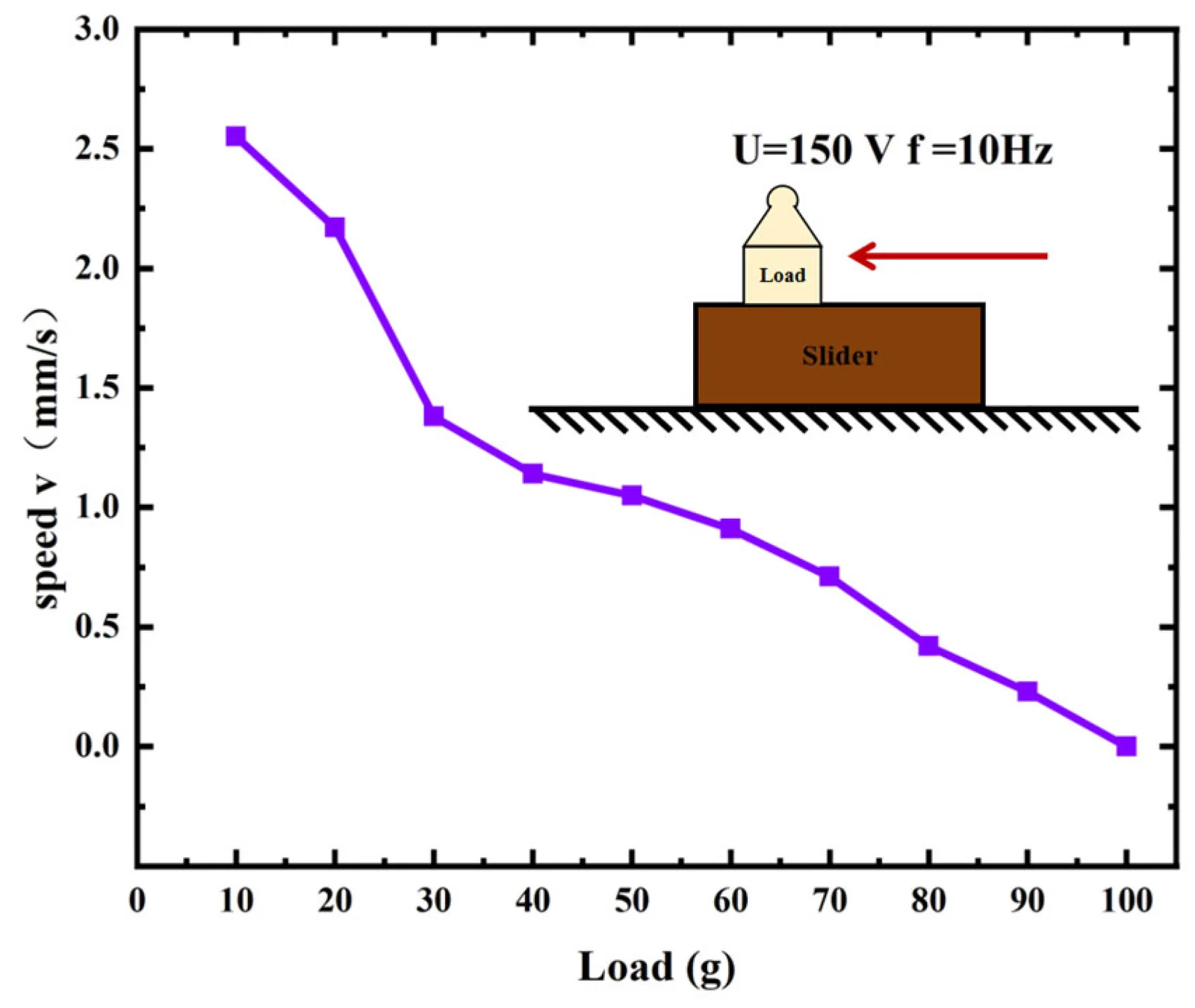
Relationship between load and speed.

The load-bearing capacity of the piezoelectric actuator in the direction perpendicular to the slider surface is also one of the important output performances. In the experiment, standard weights are placed on the slider, and the weight of the weights is used to simulate the load of the actuator. With the increase in the weight of the placed weights (increasing by 10 g each time), the bearing load also increases accordingly. The output performance of the actuator is shown in Figure 17. When the driving voltage is 150 V and the frequency is 10 Hz, the driving speed decreases gradually with the increase in the load. It is worth noting that when the bearing load reaches 100 g, the output speed is 0 μm/s, and when the load is 90 g, the output speed is 0.23 mm/s, which indicates that the designed actuator can bear a maximum load of 90 g and also shows that the positioning platform has excellent load-bearing capacity.

The output performance of the proposed piezoelectric actuator is compared with three previously reported stick-slip piezoelectric actuators of comparable planar dimensions, and the results are listed in Table 6. It can be seen that the proposed actuator has a more compact structure with a thickness of only 5 mm, which is much thinner than the 16–20 mm thickness of the referenced prototypes. More significantly, the proposed actuator delivers a maximum speed of 4.58 mm/s at a driving frequency of 100 Hz. In contrast, the compared actuators require much higher driving frequencies of 220–510 Hz to reach their peak speeds of 2.34–4.1 mm/s. This comparison clearly demonstrates that the proposed actuator can achieve high-speed driving even under low-frequency excitation. Meanwhile, the actuator exhibits a maximum load capacity of 90 g at 10 Hz, presenting favorable load-bearing performance under low-frequency operating conditions.

## 5. Conclusions

To suppress the backward motion that has a negative impact on the actuator performance and achieve the bi-directional output capability, this paper proposes a new type of stick-slip piezoelectric linear actuator and explains its driving principle and process. The finite element analysis method is used to analyze the static characteristics of the flexible mechanism. To verify the feasibility of this method, a prototype actuator is designed and fabricated for control experiments, and a control group is set up to intuitively compare with the results of active suppression. The results show that the motion mode with the backward motion suppression mechanism has a good effect. The main performance indicators of the proposed actuator under specific operating conditions are summarized as follows. Under a driving voltage of 150 V, the actuator achieves a maximum no-load output speed of 4.58 mm/s at 100 Hz and delivers a maximum load capacity of 90 g at 10 Hz. When operating at 50 Hz with the same driving voltage, the forward and reverse displacements within 1 s are 918.96 μm and 896.03 μm, respectively, with a maximum bi-directional positioning error of 5.02%. In addition, the actual displacement amplification ratio of the flexible mechanism reaches 4.738 under a 150 V driving voltage.

This study presents certain limitations. The current work only focuses on the basic output performance of the actuator at room temperature, and long-term reliability factors such as contact wear, temperature drift, humidity influence and friction coefficient variation are not systematically investigated, which may affect the output stability and service life of the device in practical applications. In addition, the load tested in this paper is the normal load perpendicular to the slider surface, and the axial thrust performance along the motion direction remains to be further verified. Furthermore, the “autonomous” backward motion suppression is achieved through the mechanical structure and fixed dual-channel timing signals without additional force feedback or closed-loop control, which still requires precise waveform timing matching rather than fully autonomous control without external signals.

## 6. Future Outlook

1. Performance gap with biological crab claws: Real crab claws have multi-joint degrees of freedom, high force-to-weight ratio and multimodal motion ability. The current prototype only realizes single-DOF linear motion with 90 g output force. To approach bionic or robot-level performance, subsequent work will optimize flexible hinge topology, adopt array PZT stacks to improve force density, and design multi-joint articulated structures to realize grasping and swinging motions.
2. Alternative piezoelectric materials (PVDF): PVDF has high flexibility and can theoretically reduce impact during reset, but its piezoelectric constant is much lower than PZT, with more severe creep and insufficient output force. For the medium-load high-precision scenario in this work, PZT remains the optimal choice. PVDF can be considered for future low-load soft bionic claw applications.
3. Development directions of bionic crab-claw actuators: Future research will expand in three dimensions: structural bionics (multi-DOF joint design), material bionics (piezoelectric composites with gradient stiffness), and control bionics (biologically inspired multi-PZT cooperative driving algorithm).
4. Long-term cyclic fatigue tests and environmental adaptability tests will be carried out to verify the service life and stability of the actuator.

## Figures and Tables

**Table 1:** Simulation parameters of 7075 aluminum alloy.

Material	Density	Young’s Modulus	Poisson Ratio
7075 AL	2810 kg/m^3^	71.7 GPa	0.33

**Table 2:** Analysis of theoretical and simulation parameters.

Working Piezoelectric Ceramics	Theoretical Displacement	Simulation Displacement	Percent Error
PZT-A	14.2 μm (Pre-tensioning direction)	14.4 μm (Pre-tensioning direction)	1.39%
PZT-A, PZT-B	39.6 μm (Output direction)	38.2 μm (Output direction)	3.66%

**Table 3:** Six-order natural frequencies.

Mode	1	2	3	4	5	6
Frequency [Hz]	379.89	379.98	1315.5	1316.9	1338.4	1340.7

**Table 4:** Theoretical, simulation, and experimental amplification ratio results.

Structure	Calcuation	FEM Analysis	Experiment
Amplification ratio	4.927	4.815	4.738
Percent Error	3.99%	1.62%	-

**Table 5:** Forward and reverse output displacement errors.

Frequency [Hz]	10	20	30	40	50
Forward displacement (μm)	321.91	607.94	761.66	871.31	918.96
Reverse displacement (μm)	333.77	577.41	753.98	845.29	896.03
Difference ratio (%)	3.68%	5.02%	1.01%	2.99%	2.49%

**Table 6:** Comparison with previous piezoelectric actuators.

Origin	Prototype Size (mm^3^)	Voltage (V)	Max Speed (mm/s)	Max Load (g)
[32]	80 × 60 × 20 mm^3^	150	4.1 (370 Hz)	70 (200 Hz)
[33]	80 × 80 × 20 mm^3^	100	2.34 (220 Hz)	380 (220 Hz)
[34]	80 × 65 × 16 mm^3^	100	3.27 (510 Hz)	-
This work	68 × 39 × 5 mm^3^	150	4.58 (100 Hz)	90 (10 Hz)

## Data Availability

All data generated or analyzed during this study are available within the Article.

## Abbreviations

The following abbreviations are used in this manuscript:

PZT	Lead Zirconate Titanate
TCF	Total Force
NPF	Non-Preload Force
